# Genetic Variation and Recent Positive Selection in Worldwide Human Populations: Evidence from Nearly 1 Million SNPs

**DOI:** 10.1371/journal.pone.0007888

**Published:** 2009-11-18

**Authors:** David López Herráez, Marc Bauchet, Kun Tang, Christoph Theunert, Irina Pugach, Jing Li, Madhusudan R. Nandineni, Arnd Gross, Markus Scholz, Mark Stoneking

**Affiliations:** 1 Max Planck Institute for Evolutionary Anthropology, Leipzig, Germany; 2 Institute for Computational Biology, Shanghai Institutes for Biological Sciences, Chinese Academy of Sciences, Shanghai, China; 3 National Drug Screening Laboratory, China Pharmaceutical University, Nanjing City, China; 4 Laboratory of DNA Fingerprinting, Centre for DNA Fingerprinting and Diagnostics, Nampally, Hyderabad, India; 5 Institute for Medical Informatics, Statistics and Epidemiology, University of Leipzig, Leipzig, Germany; University of Wisconsin, United States of America

## Abstract

**Background:**

Genome-wide scans of hundreds of thousands of single-nucleotide polymorphisms (SNPs) have resulted in the identification of new susceptibility variants to common diseases and are providing new insights into the genetic structure and relationships of human populations. Moreover, genome-wide data can be used to search for signals of recent positive selection, thereby providing new insights into the genetic adaptations that occurred as modern humans spread out of Africa and around the world.

**Methodology:**

We genotyped approximately 500,000 SNPs in 255 individuals (5 individuals from each of 51 worldwide populations) from the Human Genome Diversity Panel (HGDP-CEPH). When merged with non-overlapping SNPs typed previously in 250 of these same individuals, the resulting data consist of over 950,000 SNPs. We then analyzed the genetic relationships and ancestry of individuals without assigning them to populations, and we also identified candidate regions of recent positive selection at both the population and regional (continental) level.

**Conclusions:**

Our analyses both confirm and extend previous studies; in particular, we highlight the impact of various dispersals, and the role of substructure in Africa, on human genetic diversity. We also identified several novel candidate regions for recent positive selection, and a gene ontology (GO) analysis identified several GO groups that were significantly enriched for such candidate genes, including immunity and defense related genes, sensory perception genes, membrane proteins, signal receptors, lipid binding/metabolism genes, and genes involved in the nervous system. Among the novel candidate genes identified are two genes involved in the thyroid hormone pathway that show signals of selection in African Pygmies that may be related to their short stature.

## Introduction

The introduction of rapid, efficient, and relatively inexpensive platforms for simultaneous genotyping of hundreds of thousands of single-nucleotide polymorphisms (SNPs) has revolutionized disease-association studies, as genome-wide scans have identified many SNPs associated with complex diseases [1], [2]. One outcome of these efforts, the HapMap project [3], [4], has lead to new insights into the demographic history [5] of the three major HapMap populations (Yoruba, European, and Chinese/Japanese), as well as the identification of potential signals of recent positive selection [6]–[10]. More recently, genome-wide scans have been applied to worldwide [11]–[14], regional [15]–[17] and local [18], [19] populations, resulting in new insights into the genetic structure and relationships of human populations.

An important resource that has substantially advanced studies of worldwide genetic variation is the CEPH Human Genetic Diversity Panel (HGDP-CEPH), a collection of some 1064 cell lines from 52 worldwide populations [20], from which DNA is made available. In order to provide useful background information for ongoing studies of genome-wide variation in particular population samples in our laboratory, we decided to genotype a subset of 255 individuals from the HGDP-CEPH, consisting of 5 individuals from each of the 51 populations, for approximately 500,000 SNPs with the Affymetrix GeneChip Human Mapping 500 K Array Set. During the course of this work, genotypes became available for 938 individuals from the HGDP-CEPH, analysed for approximately 650,000 SNPs with Illumina HumanHap 650 K Beadchips [13]. The overlap between the Illumina 650 K and Affymetrix 500 K chips is 96,849 SNPs, and the availability of the Illumina 650 K genotypes thus enhances our study in two ways. First, the overlapping SNPs were used to improve the final genotype calls for the Affymetrix platform. Second, when non-overlapping SNPs between the two platforms are merged, the resulting dataset consists of over 950,000 SNPs genotyped in 250 individuals, making this the most comprehensive genome-wide scan of worldwide populations to date.

Although most analyses of the Affymetrix and Illumina gave concordant results when analysed separately, thereby justifying merging the datasets, we did identify some important differences. Our analyses of the genetic structure and relationships of worldwide populations both confirm and extend the results of previous such analyses of the HGDP-CEPH [12], [13], [21]. In addition, we modified a previous method for identifying signals of recent positive selection in genome-wide data [9], and used this method to identify many novel signals at both the individual population and regional level. Of particular interest are two genes in the thyroid hormone pathway that exhibit strong signals of local selection in Mbuti and Biaka Pygmies that may be related to the short stature of these groups.

## Results

### Worldwide Genetic Variation and Structure

We genotyped 255 unrelated individuals (five individuals from each of 51 populations; Table S1) from the HGDP-CEPH [20] for more than 500,000 SNPs with the Affymetrix GeneChip Human Mapping 500 K Array Set. During the course of this work, genotypes for about 650,000 SNPs, obtained with the Illumina Human Hap650 K Beadchips, became available for 938 unrelated HGDP-CEPH individuals [13]. A total of 250 individuals were genotyped with both platforms, and all subsequent analyses were based on 954,063 non-overlapping, merged, and filtered SNPs genotyped in these 250 individuals.

**Figure 1:**
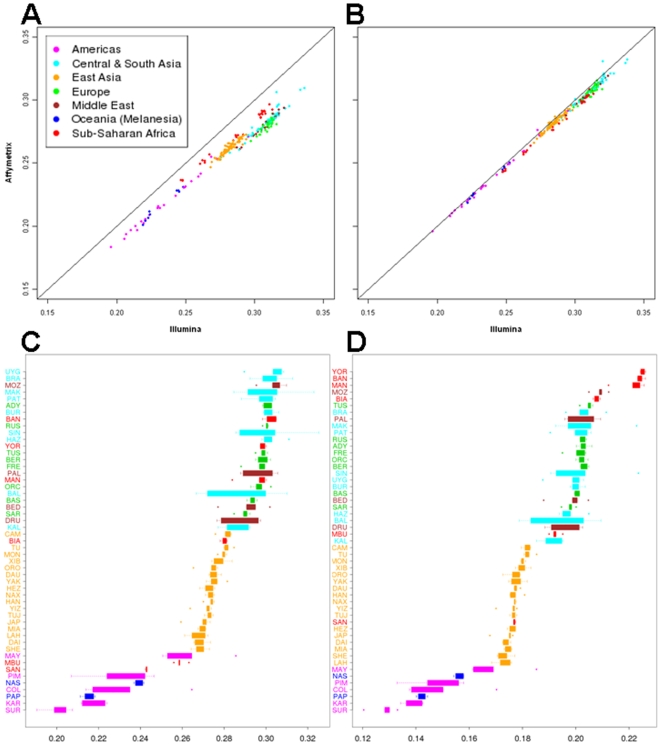
Comparison of the observed heterozygosity per individual for Illumina vs. Affymetrix genotypes. (A) For all SNPs genotyped on each platform. (B) For only those SNPs present on both platforms. (C) Box-and-whisker plot of heterozygosity values for each group, based on the entire dataset of 954,063 SNPs. (D) Box-and-whisker plot of heterozygosity values for each group, based on a pruned dataset of 220,247 SNPs in which SNPs in high LD were removed.

The observed heterozygosity for each individual was significantly higher (Mann-Whitney test, p<0.0001) for the Illumina SNPs than for the Affymetrix SNPs (Fig. 1A). In principle, this could be either because of a difference between the two platforms in how genotypes are called, or a difference in how the SNPs were ascertained. Observed heterozygosities for the 82,798 filtered SNPs in common between the two platforms are only slightly higher for the Illumina genotypes than for the Affymetrix genotypes (Fig. 1B), indicating that differences in genotype calling cannot account for the discrepancy in observed heterozygosity. Apparently, Illumina SNPs were ascertained to have a higher heterozygosity than Affymetrix SNPs.

Individual heterozygosities also change drastically when SNPs in high LD are pruned (Figs. 1C, 1D). Heterozygosity values for the sub-Saharan African, Mozabite, and Palestinian groups are increased for the pruned data (even for small, isolated populations such as San and Mbuti Pygmy), and those for some Pakistan groups with high heterozygosity values for the complete data are decreased for the pruned data. This is similar to the effect previously observed when haplotype heterozygosity values, rather than individual SNP heterozygosity values, are calculated for the HGDP-CEPH groups [12], which is not unexpected since both the pruning and the analysis of haplotypes instead of individual SNPs are intended to minimize the effect of LD. This analysis thus substantiates the well-known result that sub-Saharan African populations have the highest genetic diversity values in the world [22].

We next analyzed the genetic relationships and ancestry of individuals without assigning them to populations. First, principal component analysis (PCA) was carried out separately for the Illumina and Affymetrix SNPs, to see if the difference in observed heterozygosities between the platforms influenced the PCA results. The plots of PC1 vs. PC2 are virtually identical (Figs. S1A, S1B), although some differences in the order of particular patterns (but not the presence or absence of a pattern) appear after PC5 (Fig. S1C); PCA analysis was therefore carried out on the merged dataset of 954,063 SNPs.

We investigated the effect of LD on the PC analyses by varying the number of preceding SNPs (N_reg_) considered in a regression analysis to predict the genotypes for each SNP, and then performing PCA on the residuals of the regression analysis, as described previously [23]. In general, the results for the top PCs did not change with different values of N_reg_, except that as N_reg_ increased, decreasing amounts of the variation explained (Fig. S2), and less significant TW statistics (not shown), were associated with the top PCs. This effect of increasing N_reg_ is similar to LD pruning by removing SNPs that are closely-associated with other SNPs; for example applying a standard LD pruning in PLINK to the full dataset yields 220,247 SNPs and is similar in effect to using N_reg_>3 (Fig. S2). Given the overall small impact of LD on the PC analysis, and in order to preserve as much as possible information relevant to the demographic history of these populations, we present here the results obtained with N_reg_ = 0.

**Figure 2:**
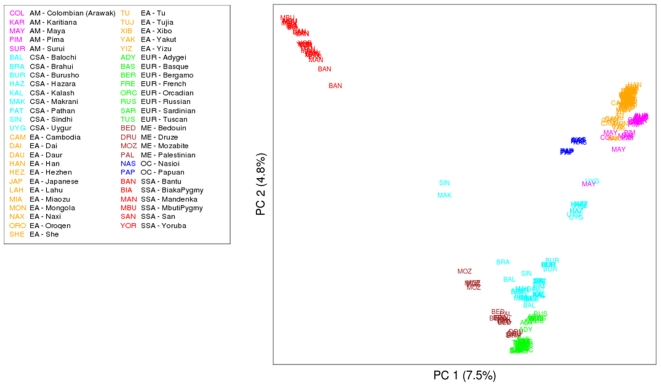
Plot of PC1 vs. PC2 for the 250 HGDP-CEPH individuals using the combined, non-overlapping set of 954,063 SNPs from the Affymetrix and Illumina platforms. Population labels are abbreviated to the first 3 letters of the population name. Regional labels are: AM = Americas, CSA = Central/South Asia, EA = East Asia, EUR = Europe, ME = Middle East, OC = Oceania (Melanesia), SSA = Sub-Saharan Africa.

**Figure 3:**
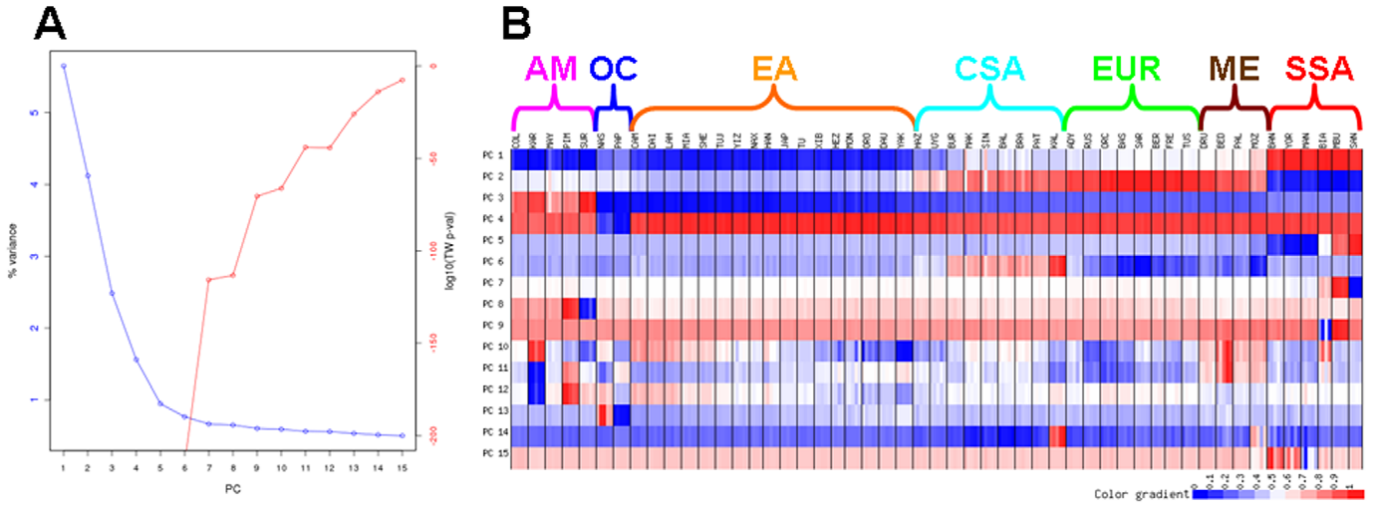
Information from additional PCs. (A) Amount of variation explained and associated statistical significance, based on the TW statistic, for the first 15 PCs. (B) Heat plot of the value of each of the first 15 PCs for each of the 51 populations. The PC values have been normalized for each PC to range from 0 to 1.

A plot of PC1 vs. PC2 (Fig. 2) reveals the same pattern observed previously in similar studies [12], [13], [21], with PC1 differentiating sub-Saharan Africans from all other individuals, and PC2 indicating an east-west spread of individuals across Eurasia. The amount of variation explained by each of the first 41 PCs is significantly greater than zero (p<0.001), according to the TW statistic [23]. However, statistical significance does not necessarily imply biological relevance; by PC15 there is a leveling-off of both the amount of variation explained and the significance of the TW statistic (Fig. 3A). We therefore focus attention on the first 15 PCs (Fig. 3B), each of which is statistically-significant at p<10^−14^ and explains more than 0.52% of the variation (Fig. 3A). There are two important aspects of human genetic history that are emphasized by these additional PCs. First is the role of migration, including dispersals out of sub-Saharan Africa and across Africa and the Eurasian landmass (PC1, PC2, and PC7), into the Americas (PC3), and into Oceania (PC4). Second is the importance of genetic structure within Africa: PC5 separates African agricultural groups (Yoruba, Mandenka, and Bantu) from African hunter-gatherers (San, Mbuti Pygmy, and Biaka Pygmy); PC6 separates the San from the two Pygmy groups; PC8 separates the Mbuti Pygmies from the Biaka Pygmies; PC11 separates the Bantu and Yoruba from the Mandenka; and PC14 separates the Bantu from the Yoruba. PCs 9, 12, 13 and 15 tend to distinguish various combinations of Karitiana from Pima and Surui, Bantu from Yoruba, and/or Nasioi from Papuan. Beyond PC15 the patterns become less clear cut, and mostly distinguish various combinations of individuals within the sub-Saharan African groups (Fig. S3). Thus, relative to the number of African groups in the sample, genetic structure within Africa has a clearly disproportionate influence on worldwide human genetic diversity.

To ensure that these patterns do not simply reflect high differentiation at a few SNPs, we removed SNPs with F_st_ values in the top 10% of the F_st_ value distribution and repeated the PC analysis. The results, based on 853,971 SNPs with F_st_ values less than 0.2126, are virtually identical up to PC9, and highly similar thereafter, to the results based on the entire data set (results not shown). Thus, the PC analysis reflects genome-wide patterns, not just a few highly-differentiated SNPs.

**Figure 4:**
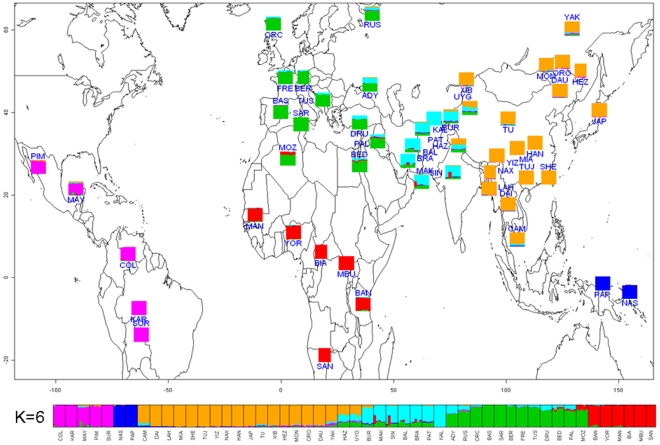
*frappe* results for K = 6. Each color indicates a different ancestry component.

We also used a maximum-likelihood method, implemented in the software *frappe* [24], to assign ancestry components to each individual without any prior assumptions about clustering of individuals into groups. In principle, the sharing of an inferred ancestry component among individuals could reflect recent admixture, ancient shared ancestry, or both. Although additional knowledge concerning the likely history of the populations involved can help distinguish among these possibilities, this is a descriptive rather than a statistical analysis. As the method does not allow inference of the most likely number of clusters (K), we ran the analysis multiple times and observed highly concordant results across multiple runs for values of K up to 6. For K = 7 and beyond different runs of the software gave different results, although continental subdivisions remained largely similar. We therefore present the results obtained with K = 6 (Fig. 4), which are largely concordant with the PC analysis and with previous such analyses of the HGDP-CEPH[13], [21]. The six clusters roughly correspond to the Americas, sub-Saharan Africa, North Africa/Europe/Middle East, Central/South Asia, East Asia, and Oceania. The Hazara (Pakistan) and Uygur (China) have roughly equal amounts of the North Africa/Europe/Middle East, Central/South Asia, and East Asia ancestry components, while all of the other Central/South Asia groups have (in addition to the Central/South Asia ancestry component) appreciable amounts of the North Africa/Europe/Middle East ancestry component (except for the Kalash). The Adygei (Caucasus), Russia, and all three Middle East groups have (in addition to the North Africa/Europe/Middle East ancestry component) appreciable amounts of the Central/South Asia ancestry component; the Bedouin and Palestinian groups (Middle East) and the Mozabite (North Africa) also have some of the sub-Saharan Africa ancestry component. One Makrani and one Sindhi individual, both from Pakistan, are identified in both the PC (Fig. 2) and *frappe* (Fig. 4) analyses as probably having recent sub-Saharan African admixture.

### Regional Population Structure and Relationships

Additional insights come from PC and *frappe* analyses conducted at the regional level. As in the worldwide analysis, *frappe* results are presented for the highest K value for which concordant results were always obtained across multiple independent runs of the software. When just the Central/South Asia, Middle East, North Africa, and European groups are analyzed, PC1 (Fig. S4A) distinguishes the Mozabite (North Africa), Middle East, and Europe groups from the Central/South Asian groups, while PC2 separates the Mozabite and Middle East groups from the Europe groups, with no overlap among individuals from the different North Africa/Middle East/Europe groups. By contrast, there is overlap among individuals from the different Central/South Asia groups; in addition, the Makrani and Sindhi individuals identified in the worldwide analysis as having experienced recent sub-Saharan African admixture are clearly differentiated by PC2. The *frappe* analysis at K = 5 (Fig. S4B) indicates ancestry components corresponding to the Mozabite, Kalash, Hazara/Uygur, other Central/South Asia, and Europe groups. The three Middle East groups have varying amounts of the Europe, Mozabite, and Central/South Asia ancestry components. The three Italian groups are alone among European groups in having low amounts of the Mozabite ancestry component, possibly indicating gene flow across the Mediterranean. The Sardinians differ from continental European groups in lacking any Asian ancestry component, while the Russians and Adygei differ from other European groups in having appreciable amounts of the Hazara/Uygur and other Central/South Asia ancestry components, respectively, indicating more gene flow and/or ancestry with these groups (Fig. S4B).

The Hazara and Uyghurs are of interest in that they consistently overlap in PC plots, even in higher PCs (Fig. S3) and share similar ancestry component profiles (Fig. 4 and Fig. S4B), which is remarkable considering that they speak very different languages and their sampling locations are separated by approximately 1500 km (Fig. 4). Although the Uyghur are often thought to exhibit European and East Asian admixture [13], [19], in the worldwide analyses the Uyghur show genetic affinities with Pakistani, East Asian, and European groups (Fig. 4). Moreover, when all Eurasian groups are analyzed, the PCA (Fig. S5A) indicates that the Hazara and Uyghur fall on an axis between East Asia (possibly Siberia) and some unknown Central/South Asian source that is either now extinct or has yet to be sampled. The *frappe* analysis at K = 4, for just Eurasia (Fig. S5B), indicates four ancestry components corresponding roughly to Europe, Central/South Asia, northern East Asia, and southern East Asia. The Hazara and Uyghur are unique among all Eurasian groups in having all four ancestry components, further indicating that their history is more complex than a simple picture of admixture between Europe and East Asia.

When just the East Asian groups are analyzed, the plot of PC1 vs. PC2 (Fig. S6A) indicates a north-south axis of genetic differentiation, from Yakuts to Cambodia, with considerable overlap of individuals from different groups. This north-south cline is also evident in the *frappe* analysis for K = 2 (Fig. S6B), which was the highest K value for which consistent results were obtained across independent runs of the software (at K = 3, sometimes Lahu were distinguished, and sometimes Japan was distinguished). This north-south distinction (also evident in the *frappe* analysis for Eurasia; Fig. S6B) is commonly observed in genetic analyses of East Asian populations [25], but it still remains unclear as to whether this is due to a single origin from the south and spread northward; a single origin from the north and spread southward; or separate origins for northern and southern East Asian groups and bi-directional migration.

When just the sub-Saharan African groups are analyzed, the three hunter-gatherer groups (San, Mbuti Pygmy, and Biaka Pygmy) are clearly differentiated in the PCA (Fig. S7A) from one another and from the three food-producing groups (Bantu, Mandenka, and Yoruba). In addition, although closely grouped, there is no overlap in the PCA between the Bantu, Mandenka, and Yoruba. The *frappe* analysis (Fig. S7B) at K = 4 completely distinguishes the San, Mbuti Pygmy, Mandenka, and Bantu from one another. The Yoruba have both the Mandenka and Bantu ancestry components, which is consistent with their geographic proximity to both the Mandenka and to the presumed ancestral home for the Bantu expansion in or near Cameroon [26]. The Biaka Pygmies have appreciable amounts of both the Bantu and the Mbuti Pygmy ancestry components, as well as (intriguingly) a smaller amount of the San ancestry component, which may indicate different interactions among hunter-gatherer groups in the past.

Finally, when just the groups from the Americas are analyzed, the PCA (Fig. S8A) clearly distinguishes all five groups from one another. The Piapoco and Curripaco (Colombia) and Maya (Mexico) show the closest affinities, even though they are from different continents. The *frappe* analysis (Fig. S8B) completely distinguishes the Pima, Maya, Karitiana, and Surui from one another, while the Piapoco and Curripaco have, in addition to their own different ancestry component, small amounts of the Maya, Karitiana, and Surui ancestry components. These results most likely reflect the role of genetic drift and/or bottlenecks in establishing detectable genetic differences among these groups with relatively small population sizes from the Americas (also reflected in their overall lower heterozygosity values, as shown in Fig. 1), as well as a more complex history for the Piapoco and Curripaco.

### Recent Local Selection

We developed a modified lnRsb method [9] to detect signatures of recent local selection in genome-wide SNP data from multiple populations (see Methods). The rationale behind this method is that SNPs in a genomic region that has experienced recent positive selection in one population will show larger extended haplotype homozygosity (EHH) values than the EHH values of the same SNPs in a population that has not experienced such selection. Since some demographic processes can produce signals in genomic data that mimic signs of selection [27], [28], we used stringent criteria to identify candidate regions of recent local selection: we calculated an lnRsb_AR_ value for each SNP, defined “top SNPs” (tSNPs) as those within the upper 1% of the distribution of lnRsb_AR_ values, and required candidate regions to have at least 25 tSNPs in a 100 kb region for the individual population comparisons, and at least 10 tSNPs in a 100 kb region for the continental regional comparisons (see Methods). We do not expect that all of these candidate regions have experienced local selection, nor does the absence of a signal in a particular population indicate that selection has not occurred in that population; however, we do expect that the candidate genomic regions identified in this study are enriched for any regions that have indeed experienced local selection [9], [29].

We also used simulations to investigate the power of our approach to detect selection and found a negligible effect of sample size on power, even for sample sizes as small as 10 chromosomes (Fig. S9). Other approaches for detecting selection either have low power with small sample sizes, e.g. iHS [30], or assume that the selected allele has been fixed, e.g. Sweepfinder [7], and hence were not used.

**Figure 5:**
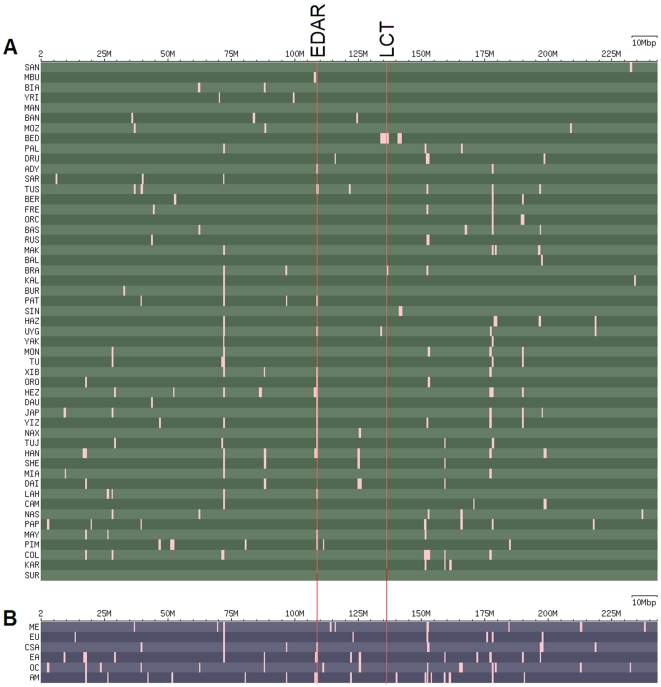
Plot of candidate regions for recent local selection on chromosome 2. Each horizontal row is a population (A) or regional group (B), with abbreviations for population names given in Table S1. The numbers across the top indicate the position (in megabases) along the chromosome, and each light box indicates a candidate region of recent positive selection. The vertical red lines indicate the position of the EDAR and LCT genes. (A) For all 51 populations. (B) For six regional groups of populations (excluding sub-Saharan Africa).

Our analysis found wide-spread signals of local adaptation at both the individual population level and the continental regional level. A list of the 25 top candidate regions indicative of positive selection in each population is provided in Table S2, and the candidate regions on chromosome 2 are depicted for each population in Fig. 5A. Many of these candidate regions overlap with one another, and in total there are 632 non-overlapping candidate regions throughout the genome across the 51 populations. Moreover, there is widespread sharing of candidate genomic regions among populations; about 33.1% (209/632) of the candidate regions detected at the population level occur in at least two different populations (Table S2), and many of these are shared across broad geographic regions.

**Figure 6:**
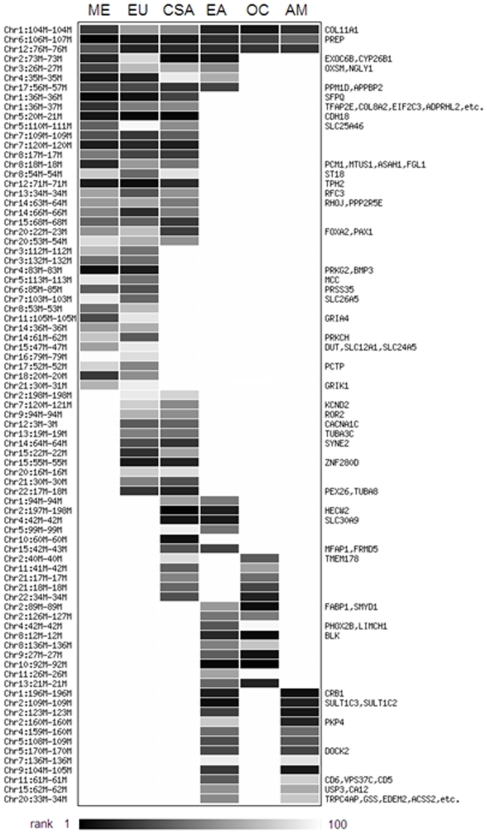
Candidates for recent local selection shared by two or more regional groupings of populations. Each row is a candidate region for recent positive selection, and each column is a regional grouping of populations. The darkness of the bar in each entry indicates the rank of that candidate region within the top 100 signals for that region (ordered by p-value), according to the scale bar at the bottom.

However, because of the stringent criteria used to identify candidate regions, and the relatively low sample size of 10 chromosomes for individual populations, some signals shared across a region may not show up in particular populations. We therefore grouped populations into regions (as shown in Fig. 2) and repeated the analysis for recent local selection. Here, we considered the 100 top ranking candidates from each regional group, since we expect in each regional group there should be collectively more candidate signals than at the population level; a list of these candidate regions is provided in Table S3 and the candidates identified on chromosome 2 in the regional comparison are depicted in Fig. 5B. The analysis at the regional level also shows that about 29.3% (115/393) of the candidates are shared across more than one region (Fig. 6). The highest number of shared candidates is found for Central/South Asia-Middle East-Europe (16), followed by Middle East-Europe (15), East Asia-America (12), Central/South Asia-Europe (11), Oceania-East Asia (9) and Central/South Asia-East Asia (5). This largely reflects the geographic relationships of the non-African regional groups, and indicates a highly correlated history of recent positive selection among the Central/South Asia, Middle East, and European groups. The high overlap in candidates between East Asia and the Americas, and East Asia and Oceania, is presumably indicative of selection events that occurred in East Asian ancestors of native Americans and of Oceanians, respectively.

Numerous genes that were previously reported to have undergone selection overlap with our candidate regions at either the population or regional level (Tables S2 and S3), including: pigmentation related genes SLC24A5 (in the Middle East and Europe regions), TYRP1 (in seven non-African populations and in the Middle East, Europe and America regions) and OCA2 (in Dai and the East Asian region); diet related genes TRPV5 and TRPV6 (in Tujia) and LCT (in Bedouin and Brahui); drug response related gene ABCB1 (in seven world-wide populations and the Central/South Asia region); sepsis related gene ASP12 (in the Middle East region), microcephaly related gene MCPH1 (in New Guinea), cation transporter gene SLC22A4 (in Mbuti Pygmy and the Central/South Asia, Europe, Middle East and America regions), and cell surface receptor gene EDAR (in Adygei, Tuscan, Pathan, Maya, Pima and eleven East Asian populations, and in the Central/South Asia, East Asia and America regions), which is important for hair and teeth development.

### GO Enrichment Analysis

To further investigate these candidate regions, we performed a Gene Ontology analysis for the genes in the candidate regions identified at the population level. The FUNC hypergeometric test was performed with 5000 random sets [31], which identified many GO groups that are significant at a false discovery rate (FDR) level of 5%. GO terms of too general definition, or those that repeated information, were removed to reduce redundancy (Table 1). These GO groups can be categorized as involving several classes of genes previously hypothesized to be subject to selection [10], [32], including: immunity and defense related genes; sensory perception genes; membrane proteins; and signal receptors. However, our analysis additionally identifies several novel GO groups related to nervous system function (Table 1), including cognition, neurological system processes, neuropeptide signaling pathway, and metabotropic glutamate, GABA-B like receptor activity (involved in synaptic transmission).

**Table 1:** GO categories significantly over-represented among candidate gene regions for recent positive selection, as identified by the FUNC analysis.

GO name	GO ID	FDR for over-representation
sensory perception of chemical stimulus	GO:0007606	0
G-protein coupled receptor activity	GO:0004930	0
olfactory receptor activity	GO:0004984	0
integral to membrane	GO:0016021	0.00E+00
signal transducer activity	GO:0004871	1.14E−04
molecular transducer activity	GO:0060089	1.14E−04
cognition	GO:0050890	0.000779879
rhodopsin-like receptor activity	GO:0001584	0.000997632
neurological system process	GO:0050877	0.0016896
MHC protein complex	GO:0042611	0.00179347
glucuronosyltransferase activity	GO:0015020	0.00303429
extracellular region	GO:0005576	0.00539895
antigen processing and presentation	GO:0019882	0.00551127
integrin complex	GO:0008305	0.00656041
neuropeptide signaling pathway	GO:0007218	0.0208835
xenobiotic metabolic process	GO:0006805	0.0401682
metabotropic glutamate, GABA-B-like receptor activity	GO:0008067	0.0406189
sulfate transport	GO:0008272	0.0423062

FDR, false discovery rate.

### Novel Candidates in African Pygmies

**Figure 7:**
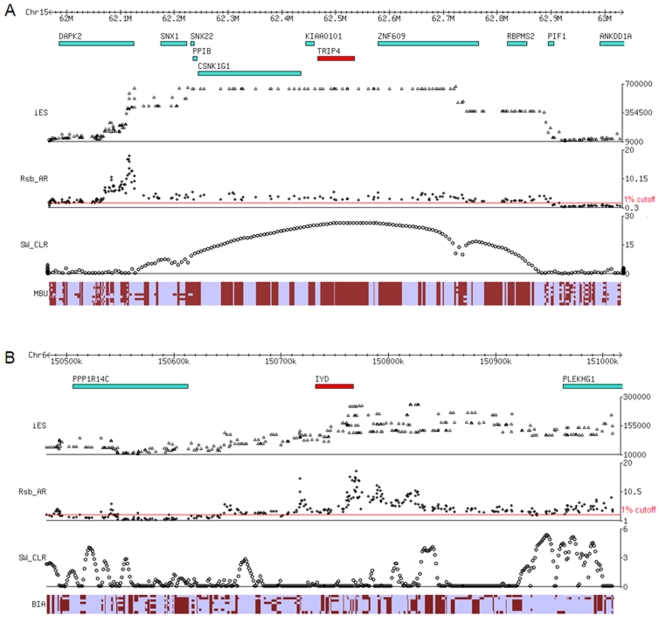
Signals of selection for thyroid hormone pathway genes in African Pygmies. (A) 1 Mb region around TRIP4 in Mbuti Pygmies. (B) 500 Kb region around IYD in Biaka Pygmies. In each panel, the top part is the region surrounding the candidate gene with the position of genes indicated, followed by the distribution of the iES, Rsb_AR_, and Sweepfinder [7] statistics, followed by a diagram of the haplotypes observed in either Mbuti (A) or Biaka (B) Pygmies, where each row is a haplotype, each column is a SNP, and the light vs. dark shading indicates the alternative alleles for each SNP.

Of particular interest are two genomic regions that show signs of selection in the African Pygmy groups that may be related to their short stature. In the genomic region Chr15:62,071,285-62,735,847 in Mbuti Pygmies, there is an obvious increase in EHH and an absence of genetic diversity, and this signal of positive selection ranks as the second strongest in Mbuti Pygmies (Fig. 7A). A model-driven, composite likelihood ratio (CLR) test based on examination of the local allele frequency spectrum was applied to this region to estimate the center of selection [7], and revealed that the TRIP4 gene sits at the peak of the likelihood ratio values (Fig. 7A). TRIP4 is a thyroid hormone receptor interactor that interacts and activates thyroid receptor in the presence of thyroid hormone. While Biaka Pygmies do not share this signal of selection, there is another strong candidate region of selection (ranking seventh) that includes a gene closely related to thyroid hormone production. This gene is iodotyrosine dehalogenase 1 (IYD), and is near the maximum lnRsb_AR_ value in the genomic region chr6:150,648,034-151,372,379 (Fig. 7B). IYD catalyzes deiodination of mono- and di-iodotyrosine (two byproducts of thyroid hormone metabolism), and facilitates iodide salvage in the thyroid gland. Analysis of these two candidate regions in just the Illumina SNP data for the HGDP-CEPH [13], which consists of more individuals but fewer SNPs, reveals that TRIP4 is still a strong candidate region for recent positive selection in Mbuti Pygmies, but while there is still an extended EHH signature for IYD in Biaka Pygmies, it is no longer among the top 25 candidate regions. This discrepancy may be due to the smaller number of individuals in our study, or the smaller number of SNPs in the Illumina SNP data in the candidate region.

The Mbuti and Biaka Pygmy groups live in inland tropical forests with limited access to iodine-rich foods. Previously it was found that in the iodine-deficient Ituri Forest, where Pygmy and Bantu groups live in association with each other, the prevalence of goiter in the Bantu is 42.9%, compared to just 9.4% in Pygmies [33]. This observation, coupled with the strong signals of selection for TRIP4 and IYD, suggests that Pygmies may have adapted to their iodine-deficient environment by genetic changes in the thyroid hormone pathway. Moreover, these genetic changes may also be responsible for short stature, as discussed in more detail below.

## Discussion

### Genetic Variation and Population Structure

Our comparison of SNP genotypes obtained with two different genotyping platforms for the same set of individuals indicates that while much of the information captured by these platforms is similar, there are nonetheless important differences. The most striking difference is in the heterozygosity per individual (Fig. 1A); for every individual the observed heterozygosity was higher for the Illumina SNPs than for the Affymetrix SNPs, and the overall average heterozygosity for the Illumina SNPs was 0.287, significantly higher than the overall average heterozygosity of 0.265 for the Affymetrix SNPs. Although the Illumina software tends to call more heterozygotes than the Affymetrix software (Fig. 1B), this difference alone does not account for the observed discrepancy in heterozygosity values for the two platforms. Instead, it appears that the Illumina SNPs were ascertained to have higher heterozygosity values than the Affymetrix SNPs. To be sure, both platforms are biased towards detecting SNPs with high minor allele frequencies, as can be seen in the fact that heterozygosity values for both platforms are much higher than is observed in resequencing studies. For example, we calculate from a previous resequencing study [34] of Hausa, Han Chinese, and Italians observed heterozygosities of 0.15, 0.11, and 0.12, respectively, which are much lower than the heterozygosity values obtained with the SNP genotyping platforms for comparable populations. Moreover, the SNPs on both platforms overestimate heterozygosity in European populations (and those of related ancestry) relative to other populations (especially sub-Saharan Africa), as can be seen in Figs. 1A and 1C. However, when pruning of SNPs in high LD is carried out, then the familiar pattern of overall greatest genetic diversity in sub-Saharan Africa is obtained with the genome-wide SNP data, in spite of the ascertainment bias (Fig. 1D).

The merged dataset of non-overlapping, filtered SNPs from both the Illumina and Affymetrix platforms consists of nearly 1 million SNPs, making this the largest genome-wide scan of worldwide human populations. Although the genetic structure (PC and *frappe*) analyses at both the worldwide and regional levels largely recapitulate previous such analyses of the HGDP-CEPH with fewer genetic markers [12], [13], [21], [35], there are three important insights that deserve more attention. First, the first PCs emphasize the role of migration and dispersal in human history, including: dispersal out of Africa, spread across Eurasia, entry into the Americas, and entry into Oceania. Indeed, the order of the PCs closely corresponds to the relative timing of these events, with the exception that the initial colonization of Near Oceania (i.e., Australia and New Guinea) is generally considered to have occurred around 40,000–50,000 ybp [36], before the initial colonization of the Americas at not more than 15,000 ybp [37], although the more recent migration of Austronesian-speakers to Oceania has had a large genetic impact on coastal New Guinea and island Melanesian populations [38], [39]. To be sure, spatial patterns observed in PC plots may reflect isolation by distance rather than migration [40]. Nonetheless, humans did spread out of Africa, across Europe and Asia, and into the Americas and the Pacific via migrations, and this close correspondence between the order of the PCs and major human migrations suggests that there may be information in the PC analysis about the timing of such migrations (possibly coupled with the effective size of the migrating population), a possibility that we are currently exploring further.

Second, many of the subsequent statistically-significant PCs (Fig. 3 and Fig. S3) distinguish among various combinations of the sub-Saharan African groups (or among individuals within such groups), despite the fact that there are only six such groups in the analysis. This disproportionate impact of structure within sub-Saharan Africa on analyses of worldwide genetic diversity clearly emphasizes both the importance of such structure and the great need for further in-depth genetic characterization of sub-Saharan African populations [22]; we would hardly expect that these six groups encompass all of the genetic diversity in sub-Saharan Africa.

Third, previous analyses of European genetic diversity have shown that there is a surprisingly good correspondence between the genetic and geographic relationships of individuals [15], [40]. To what extent does this hold for other regions of the world? The inter-cluster PC distances are significantly correlated with the geographic distances between groups for the worldwide (p = 0.011), Europe/Middle East/Central-South Asia (p<0.00001), and East Asia (p = 0.001) comparisons, but not within the Americas (p = 0.201) or sub-Saharan Africa (p = 0.43). And while the sampling of individuals and populations in the present study is not of the same depth as in the previous studies of European diversity, nonetheless the regional PC analyses (Figs. S4, S5, S6, S7, S8) indicate that there is no overlap of individuals from different groups (despite the close clustering of some groups) from within sub-Saharan Africa, the Americas, or North Africa/Europe/Middle East. However, within Central/South Asia and within East Asia some overlap does occur among individuals from different groups. Thus, the previous findings based on European populations are not universal, and it would be of interest to explore further those cases where individuals from different groups overlap in the PC analysis (i.e., does this reflect recent common ancestry of the groups, recent admixture, or some other factor?).

### Recent Positive Selection

Previous work on recent positive selection in human populations focused primarily on single representatives from each of the three major continental regions, namely Africa (African Americans/Yoruba), Europe (European Americans/Central Europeans) and East Asians (Han Chinese and Japanese) [8]–[10], [32]. The genotyping of the HGDP-CEPH panel provides a much more comprehensive and detailed analysis of worldwide human population structure, and it is therefore of interest to determine if the previously observed signals of recent positive selection also appear in these additional populations.

Recently, another study [30] searched for signals of positive selection in the previously-generated genotype data [13] for the HGDP-CEPH. In this study, several genome-wide scan methods, including iHS [10], XP-EHH [8] and F_st_ were used to identified signals of positive selection mainly at the continental region level. Since the lnRsb method in our study is similar to the XP-EHH method and is related to the iHS method, both used in the previous study [41], we expect to see some overlap among our candidates and those identified previously. Indeed, for the continental region groups defined similarly between the two studies (i.e., the Middle East, Europe, East Asia, Oceania, and America), substantial proportions of the iHS candidates (21.4%–51.2%) and XP-EHH candidates (46.6%–63.0%) overlap with our lnRsb candidates (all p-values ∼0). This degree of overlap is greater than that usually observed among genome-wide selection studies [42]. The strong consistency between our results and the previous study, based on partly different methods, individuals, and SNPs, supports the reliability of the candidate regions. In addition, our study provides more detailed analyses at the individual population level than previous such studies.

As indicated above, we do re-capture many previously-inferred signals of positive selection, some of which are quite strong signals shared across many populations and multiple geographic regions. For example, this is the case for EDAR (Fig. 5), TYRP1 and ABCB1 (Tables S2 and S3). However some previously-inferred candidate genes show signals of recent positive selection at the regional level but less so within individual populations, such as SLC24A5, OCA2, CASP12 and SLC22A4 (Tables S2 and S3). Other previously-identified candidates, such as TRPV5/TRPV6 and LCT, do not stand out at either the regional or the individual population level.

There are three potential explanations for the failure of our analyses to detect previously-identified candidates. First, the sample sizes in our study may be too small to have sufficient power to detect signals of selection. However, the power analysis (Fig. S9) indicates that sample sizes of even just 10 chromosomes have nearly the same power as larger sample sizes. Moreover, the high degree of overlap between our candidates and those identified in a previous analysis of the HGDP-CEPH [41] suggests that low power is not an issue. Second, some signals detected previously may be out-ranked by novel signals, which are of higher significance when considering the additional populations included in our analyses; we discuss some of these novel signals below. Third, some previously-identified signals might represent population-specific signals rather than more widespread regional signals. One such example is LCT. The LCT gene was previously shown to have undergone selective sweeps in European and sub-Saharan African populations that rely on milk-drinking [43], [44]. However, both our study and the previous study [41] of the HGDP-CEPH fail to identify LCT as a candidate gene for recent positive selection in either the European regional group or in any of the individual European populations (with the exception of Orcadians in our study). This suggests that either overall European population diversity is not well-represented by the HGDP-CEPH (i.e., there is a lack of North European groups, where the strongest signals for selection on LCT would be expected), or that genes previously studied in a limited context for recent positive selection should now be re-examined in a worldwide population context.

Some of the signals of recent positive selection identified in this study are shared among many populations and/or regions. It is expected that populations with more recent common ancestry would share the same past genetic adaptations, and hence the same candidate regions. This is consistent with the observations of many overlapping signals across the Middle East, Europe, and Central/South Asia, and between East Asians and the Americas or Oceania (Fig. 6). However, another possible explanation is convergent adaptation reflecting similar selection pressures, as has been observed at LCT in European and some sub-Saharan milk-drinking groups [44], and hypothesized for TRPV6 in European and East Asian groups [45]. To distinguish between recent shared ancestry vs. convergent adaptation as possible explanations for shared signals of recent positive selection, further study of the underlying haplotype structure and age of the onset of selection would be required.

The GO analysis identified several classes of genes previously hypothesized to be subject to selection [10], [32], including: immunity and defense related genes; sensory perception genes; membrane proteins; and signal receptors. Given that these classes of genes are involved in how humans interact with their environment, especially in terms of pathogens and diet, it is not unexpected that they would be over-represented among candidate regions for recent positive selection. However, our analysis additionally identifies several novel GO groups related to nervous system function (Table 1). Although further work is necessary to confirm the hypothesized signals of recent positive selection at genes in these GO groups, we speculate that these observed signals of selection may be related to how different human groups interact behaviorally with their environment and/or with other human groups.

### Novel Candidates

In addition to the previously-identified candidate genes mentioned above, many novel candidates were identified (Tables S2 and S3); we briefly describe the most noteworthy of these. The COL11A1 gene is identified as a candidate for recent positive selection in 22 non-African populations and all six of the non-African regions (Tables S2, S3). This gene maps to the region Chr1:103114611-103346640 and encodes an alpha chain for type IX collagen. This gene is essential for cartilage tissue development and mutations in it have been associated with Stickler syndrome type III and Marshall syndrome [46], which are characterized by hearing loss and bone and joint defects. Since cartilage makes up much of the soft tissue of the face, it is tempting to speculate that this gene may play a role in facial and/or physical differences among groups. Another region (chr1:153067871-153352772), which is the top-ranking candidate in East Asia and in the Han, Japanese, and Naxi groups, and the third ranking candidate in Xibo, contains the SHC1 and KCNN3 genes. SHC1 encodes a signaling and transforming protein that couples various growth factors into the signaling pathway, and is associated with stress responses and increased lifespan in mammals [47]. KCNN3 encodes a small conductance calcium-activated potassium channel that is thought to regulate neuronal excitability, and polymorphisms in KCNN3 have been associated with several neural disorders, including schizophrenia [48]and anorexia nervosa [49]. Another strong signal of selection is associated with a cluster of β-defensin genes that map to the region Chr8: 10917927-11894726. This is one of the most widespread signals of selection in non-African populations, with18 populations across different regions sharing this signal, and it is the top-ranking candidate region in Central/South Asia. The β-defensins are a group of small, secreted proteins that are active against bacteria, fungi and many viruses. Strong signals of selection at this locus hence might suggest adaptation to local pathogens. A high-ranking candidate region in the Maya, Hazara, and several East Asian populations with nomadic origins (including Yakut, Mongola, Oroqen, Xibo and Hezhen) is Chr5:70800637-71275894, centered around the CARTPT gene. This candidate region also ranks high in several regional groups, including Central/South Asia, Middle East, Europe, East Asia, and the Americas. CARTPT encodes the cocaine- and amphetamine-regulated transcript protein Cart. It is a brain-located satiety factor that serves as an endogenous psychostimulant and plays an important role in energy homeostasis, feeding, and reward and stress responses [50]. Given the widespread signals of selection in most non-African regional groups, we speculate that adaptations influencing this gene might have played an important role in the early migrations of human populations.

It should be noted that not all of the top-ranking candidates contain genes. For example, the region Chr9:38623652-38761831 is the top-ranking candidate region in eight populations (Bedouin, Druze, Adygei, Basque, Xibo, Daur, Lahu and Pima) and two regions (Middle East and Europe), but contains no genes. Several uncharacterized transcripts are within this region; these transcripts or other potentially functional components within this region might be interesting targets for further investigation.

### Novel Candidate Genes for Short Stature in African Pygmies

The short stature of Pygmy groups around the world has long intrigued anthropologists [51], [52]. It is generally accepted that their small body size is a result of genetic adaptation; however, which genes were selected, and the nature of the underlying selective force(s), remain unknown. The various hypotheses proposed include adaptations to food limitation, thermoregulation, mobility in the forest, and/or short lifespan [52]. A recent study of the HGDP-CEPH populations identified a signal of selection in the insulin growth factor signalling pathway in Biaka Pygmies, which might be associated with short stature, but this signal was not shared with Mbuti Pygmies [41]. By contrast, we found strong signals for selection in both African Pygmy groups at two genes involved in the iodide-dependent thyroid hormone pathway: TRIP4 in Mbuti Pygmies; and IYD in Biaka Pygmies (Fig. 7). Intriguingly, a previous study [33] found a significantly lower frequency of goiter in Efe Pygmies (9.4%) than in Lese Bantu farmers (42.9%). The Efe and Lese live in close proximity to one another in the iodine-deficient Ituri Forest and share similar diets. Moreover, the frequency of goiter in Efe women living in Bantu villages was similar to that of Efe women living in the forest, and the frequency of goiter in offspring with an Efe mother and a Lese father was intermediate between that of Efe and Lese [33]. These observations suggest that the Efe have adapted genetically to an iodine-deficient diet; we suggest that the signals of recent positive selection that we observe at TRIP4 in Mbuti Pygmies and IYD in Biaka Pygmies may reflect such genetic adaptations to an iodine-deficient diet. Furthermore, alterations in the thyroid hormone pathway can cause short stature [53], [54]. We therefore suggest that short stature in these Pygmy groups may have arisen as a consequence of genetic alterations in the thyroid hormone pathway.

If this scenario is true, then there are two important implications. First, this would suggest that short stature was not selected for directly in the ancestors of Pygmy groups, but rather arose as an indirect consequence of selection in response to an iodine-deficient diet. Second, since different genes in the thyroid hormone pathway show signals of selection in Mbuti vs. Biaka Pygmies, this would suggest that short stature arose independently in the ancestors of Mbuti and Biaka Pygmies, and not in a common ancestral population. Moreover, most Pygmy-like groups around the world dwell in tropical forests [52], and hence are likely to have iodine-deficient diets. The possibility that independent adaptations to an iodine-deficient diet might therefore have contributed to the convergent evolution of the short stature phenotype in Pygmy-like groups around the world deserves further investigation.

In conclusion, obviously much more work is needed to verify the potential signals of selection at the thyroid hormone pathway genes in Mbuti and Biaka Pygmies, as well as the many other novel and intriguing signals of selection identified in this study. Nevertheless, the results of our study, and similar such studies [30], provide a rich source of novel hypotheses concerning the role of genetic adaptations and recent positive selection in human evolution that deserve further investigation. The value of assessing genome-wide data from additional worldwide populations for signals of recent positive selection is thus amply demonstrated.

## Materials and Methods

### Samples

We selected a subset of 255 individuals from the HGDP-CEPH (Table S1) for genotyping by choosing at random five unrelated individuals based on the H952 set [55] from each of 51 populations (all five Bantu individuals are from Kenya, so no South African Bantu were included). We also genotyped two samples as positive controls: Affymetrix Reference Genomic DNA 103 and HapMap #NA06985 CEPH/UTAH Pedigree 1341, for which Affymetrix 500 K consensus genotypes are available. We downloaded genotype data obtained with Illumina Human Hap650K Beadchips [13]for 250 of the HGDP-CEPH individuals that we analyzed (http://hagsc.org/hgdp/files.html).

### Genotyping and Quality Control

The Affymetrix GeneChip Human Mapping 500 K Array Set is comprised of two arrays; one uses the Nsp I restriction enzyme (262,314 SNPs), while the second uses Sty I (238,354 SNPs). Briefly, total genomic DNA (250 ng per array) was digested with each restriction enzyme, ligated to adaptors, and amplified using a generic primer that recognizes the adaptor sequence. The amplified DNA was then fragmented, labeled, and hybridized to oligonucleotide probes attached to the surface of each array. Array batches from different enzyme fractions were processed on different days in order to reduce the risk of cross-contamination. Each array was scanned using the GeneChip Scanner 3000 with the High-Resolution Scanning Upgrade. The cell intensity files were analyzed using GeneChip Analysis Software (GTYPE) v4.1 and Genotyping Console Software v2.1.

The Dynamic Model (DM) mapping algorithm integrated into the GTYPE software assigned the genotype call for each SNP, and was primarily used to perform single-array quality control (QC). All runs of the DM algorithm were performed at default settings. On average, the DM SNP call rate was 93.8% (range 87.0 – 98.1%) for the Nsp I arrays, and 92.9% (range 87.1–98.3%) for the Sty I arrays. Call and discrimination rates of the additional QC Modified Partitioning Around Medoids algorithm (MCR and MDR) did not differ over ∼20%, indicating no contamination or sample mix-up. The reported DM gender calls for each array matched the gender described in the HGDP-CEPH panel information for each sample, with one exception. For sample HGDP_01239 (Hezhen, China) the reported DM gender call (male) for both the Nsp I and Sty I arrays disagreed with the gender of the HGDP-CEPH panel information for that sample (female); however genotypes from another laboratory agreed with our gender call [55]. In order to create a virtual 500 K set combining the same sample data from the two different arrays, the Affymetrix sample mismatch report tool was run, confirming for all 255 samples that the same sample was genotyped in each paired Nsp I and Sty I array.

To assess the reliability of the genotyping experiments for the positive controls, the percentage of agreement (i.e., genotype concordance) and unweighted Cohen's Kappa statistic (i.e., percentage of agreement above and beyond chance alone) were calculated using an R script. Genotypes of the Affymetrix Reference Genomic DNA 103 were compared with the consensus genotypes provided by Affymetrix. Genotypes of the HapMap #NA06985 CEPH/UTAH Pedigree 1341 were compared with the consensus genotypes obtained from the HapMap website. Genotypes for these comparisons were obtained by the Affymetrix Bayesian Robust Linear Model with Mahalanobis (BRLMM) distance classifier; all missing data were excluded. The concordance for the genotypes from both Nsp I and Sty I arrays combined together for the Affymetrix Reference Genomic DNA 103 was 98.5%, and for the HapMap #NA06985 CEPH/UTAH Pedigree 1341 was 99.4%, while the calculated Kappa values were 0.98 and 0.99 respectively for each positive control sample.

### Genotype Calling

Genotypes used for further analysis were called with the CHIAMO algorithm (http://www.stats.ox.ac.uk/~marchini/software/gwas/chiamo.html), which has been showed to outperform the standard Affymetrix BRLMM algorithm in several respects [2]. First, Affymetrix CEL (cell intensity) files for each sample were normalized using the Cel Quantile Normalisation program (http://www.wtccc.org.uk/info/software.shtml). We then investigated genotype-calling accuracy for several parameter sets for the CHIAMO program (Fig. S10), by comparing the called genotypes for each parameter set to previously-published genotypes [13] for the same samples but based on the Illumina HumanHap 650 K Beadchips, for the 96,849 SNPs that overlap between the Affymetrix and Illumina platforms. Using allele frequency priors based on HapMap population samples improved call rates in most cases, and there was better overall concordance with the Illumina calls (Fig. S10); however we also occasionally observed a regional bias in terms of concordance with the Illumina calls, which we are investigating further (M. Bauchet, D. L. Herraez, and M. Stoneking, unpublished data). Therefore, the following CHIAMO parameter set, which minimized the discrepancy in genotype calls between the two platforms in an unbiased fashion, was used to call the genotypes: -gz -max1 -max2 -nmax 200 -n 0 -b 0 (which is the same as used in a previous study [2] except for the omission of allele frequency priors). We obtained CHIAMO calls for 488,756 autosomal SNPs.

### Filtering and Merging Datasets

We then performed filtering; first, in order to avoid inter-population biases in missing data, we defined a threshold of at least three genotypes called per population sample. This discarded 53,235 SNPs in total. We also discarded rs41388745 as it was mapped to both chromosome 1 and 7. Finally, by defining a threshold of 1% for global Minor Allele Frequency (MAF) of a SNP, we excluded an additional 18,875 SNPs, leaving a total of 416,644 filtered autosomal SNPs for further analyses. These data are available at http://bioinf.eva.mpg.de/download/hgdpceph.affy500k.tgz. We decided not to remove SNPs based on Hardy-Weinberg (HW) disequilibrium because there is low power to detect HW deviations with the sample sizes in this study, and moreover a comparable study showed that the impact of removing such SNPs was negligible [13].

Merging the Affymetrix and Illumina datasets was performed with the PLINK software v1.05 [56] (http://pngu.mgh.harvard.edu/purcell/plink). The 84,967 filtered SNPs in common were merged following the consensus method, i.e. missing calls in one of the datasets was filled from the other dataset, and mismatches between datasets resulted in a missing call in the merged dataset. When the initial filter was re-applied to this set of SNPs in common, 2,169 SNPs were subsequently eliminated, yielding a final marker set of 954,063 SNPs. PLINK was also used to prune SNPs in high LD, using the following settings: -indep 50 5 2, resulting in a dataset of 220,247 SNPs.

### Population Structure Analyses

We performed principal component analysis (PCA) with the *smartpca* program (http://genepath.med.harvard.edu/~reich/Software.htm) from the EIGENSOFT package [23], with the default settings except for numoutlieriter = 0 (no outlier pruning) and nsnpldregress = 0. The percentage of the variance explained and the Tracy-Widom statistic were calculated for each principal component (PC). To examine the effect of LD on the PCA, we investigated the effect of the parameter ‘nsnpldregress’, which changes the input to PCA to be the residual of a regression involving N_reg_ previous SNPs, according to physical location.

We also used the maximum likelihood (ML) algorithm implemented in the *frappe* software [24] (http://smstaging.stanford.edu/tanglab/software/frappe.html) to estimate individual affinities for a pre-determined number of clusters. The MaxIter setting was set to 10,000 so that the convergence criterion was always met (likelihood increase since last output less than one point per step), and we performed at least four independent runs per set of populations.

### Recent Local Selection

To identify candidate regions that have experienced recent local selection (i.e., positive selection in some, but not all, populations), we modified the lnRsb approach [9] in order to apply it to multiple populations. First, the extended haplotype homozygosity for sites (EHHS) for each SNP in each population was determined until the EHHS decayed to 10%, then the integrated EHHS was calculated and log transformed into ln(iES) values. A reference ln(iES) distribution was then formed by averaging the ln(iES) values across the six African populations, with the expectation that this should approximate the ancestral ln(iES). This results in the African-referenced lnRsb_AR_ value for each SNP in each population, that is comparable to the original lnRsb [9]:
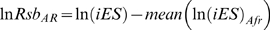



To evaluate the signals that are shared among populations, we merged the genotype data into the seven geographic regions (Africa, Europe, Mideast, Central/South Asia, East Asia, Oceania, and the Americas) suggested by previous studies [13]. The ln(iES) values for non-African regions were then compared to that of Africa:
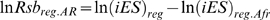



In the case of individual populations, the SNPs that carry the highest 1% lnRsb_AR_ values in each population were used to define candidate regions if there were 25 or more such top SNPs (tSNPs) within 100 kb of each other. For the analysis of the six non-African continental regions, since there are many fewer groups in the analysis, the minimum number of tSNPs required for a candidate region was relaxed to 10, while the other conditions were the same as for the individual population tests. The tSNPs were ranked across the genome in each population and the mean rank of the top 20% tSNPs in each candidate region is used as the relative signal strength of that candidate region. Candidate regions were ordered according to this score and the top 25 candidate regions were determined for each population, and the top 100 candidate regions were determined for each regional grouping of populations.

To evaluate the power of this approach to detect recent positive selection with the relatively small sample sizes (10 chromosomes per individual population) in this study, simulations were carried out. Forward simulations were carried out with the program FREEGENE (http://www.ebi.ac.uk/projects/BARGEN/download/FREGEN/, [57]) assuming three populations and demographic parameters previously inferred for Africans, Europeans, and Chinese [5]. We simulated 2 Mb regions, with SNP ascertainment as described previously [9]; an allele with a given selection coefficient was added to the Chinese population at a random position in the region and at a random time between the present and the divergence of the Chinese from the other populations. The lnRsb statistic was calculated for the SNPs within the central 1 Mb in each region, to allow sufficient decay of the EHH. Under neutrality, the top 1% SNPs are called tSNPs, and the significant cutoff value for the ratio of tSNPs in each region was determined, given a type I error rate of 0.5%. The same cutoff and tSNP criteria were then applied to the selection scenarios to evaluate the power to detect selection. For each neutral scenario 1000 simulations were carried out, while for each selection scenario 200 simulations were carried out.

### Gene Ontogeny Analysis

For the gene ontogeny (GO) analysis we used the FUNC program [31]. This method utilizes hypergeometric tests to determine the GO terms for which there are significantly more genes overlapping with the candidate regions of selection than expected under the null hypothesis of no association. We used the web version of the FUNC program (http://func.eva.mpg.de/doc/func.html) and carried out 5000 iterations to achieve stable results.

## Supporting Information

Figure S1 Comparison of PCA for the same 250 individuals genotyped on the Affymetrix vs. Illumina platforms. (A) Plots of PC1 vs. PC2. (B) The percent variation explained and the p-value of the TW statistic for the first 40 PCs. (C) Values of the first 40 PCs.
(0.35 MB TIF)

Figure S2 Effect of the number of preceding SNPs (Nreg) used to predict SNP genotypes in the regression analysis on the percent variation explained by the first 10 PCs. Also shown is the effect of pruning SNPs that are in high LD.
(0.12 MB TIF)

Figure S3 Analysis of additional PCs for the worldwide populations. (A) Amount of variation explained and associated statistical significance for the first 45 PCs. (B) Heat plot of the values of the first 45 PCs. The PC values have been normalized to range from 0 to 1.
(0.44 MB TIF)

Figure S4 Regional analysis of the Europe, North Africa, Middle East, and Central/South Asia groups. (A) Plot of PC1 vs. PC2. (B) *frappe* results for K = 5.
(0.19 MB TIF)

Figure S5 Regional analysis of the Europe, Middle East, Central/South Asia, and East Asia groups. (A) Plot of PC1 vs. PC2. (B) *frappe* results for K = 4.
(0.24 MB TIF)

Figure S6 Regional analysis of the East Asian groups. (A) Plot of PC1 vs. PC2. (B) *frappe* results for K = 2.
(0.17 MB TIF)

Figure S7 Regional analysis of the sub-Saharan African groups. (A) Plot of PC1 vs. PC2. (B) *frappe* results for K = 4.
(0.12 MB TIF)

Figure S8 Regional analysis of the groups from the Americas. (A) Plot of PC1 vs. PC2. (B) *frappe* results for K = 5.
(0.12 MB TIF)

Figure S9 Effect of sample size on the power of the lnRsb analysis to detect a positively-selected allele. For each sample size (n = number of chromosomes), the fraction of simulations (y-axis) in which a selected allele of a given selection coefficient (x-axis) was detected as a candidate region of selection, according to the criteria described in the Methods section, is plotted.
(0.06 MB TIF)

Figure S10 Effect of different parameter values on genotype calls obtained with the CHIAMO algorithm. Shown are the mean values (in percent) for heterozygous and homozygous mismatches between the Affymetrix and Illumina platforms, no calls for either or both platforms, and the sum of all mismatches and no calls. These values are given for the BRLMM algorithm for all chromosomes, and for two different parameter sets and various threshold values for the CHIAMO algorithm, for either just chromosome 22 or for all chromosomes. The threshold value is the maximum value for the total fraction of missing genotypes allowed; CHIAMO Par 1 is the WTCCC parameter set (-max1 -max2 -nmax 200 -n 0 -b 0 -f freqfile) and Par 2 is identical to Par 1 except without the -f option.
(0.09 MB TIF)

Table S1 List of the 255 HGDP-CEPH individuals genotyped in this study, including ID, population, location, region, population code, gender, heterozygosity for the Affymetrix SNPs, and heterozygosity for the Illumina SNPs.
(0.15 MB XLS)

Table S2 List of the candidate regions for recent positive selection identified in the population-level analyses, including the chromosome, start and end positions, HGNC gene ID, and populations (and signal rank) for which a positive signal was obtained.
(0.10 MB XLS)

Table S3 List of the candidate regions for recent positive selection identified in the regional-level analyses, including the chromosome, start and end positions, HGNC gene ID, and regions (and signal rank) for which a positive signal was obtained.
(0.07 MB XLS)
