# Supplementary figures and images for: Genetic Variation and Recent Positive Selection in Worldwide Human Populations: Evidence from Nearly 1 Million SNPs
Source: PLoS One. 2009 Nov 18;4(11):e7888. doi: 10.1371/journal.pone.0007888 (PMC2775638; doi:10.1371/journal.pone.0007888)

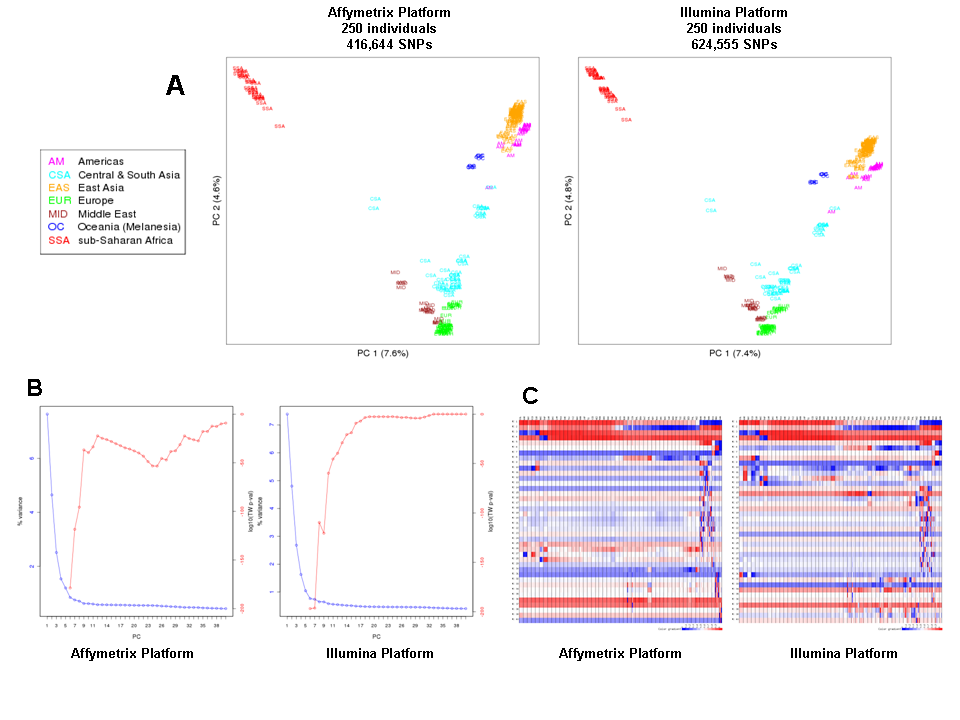

Supplement: Figure S1 — Comparison of PCA for the same 250 individuals genotyped on the Affymetrix vs. Illumina platforms. (A) Plots of PC1 vs. PC2. (B) The percent variation explained and the p-value of the TW statistic for the first 40 PCs. (C) Values of the first 40 PCs. (0.35 MB TIF) [file pone.0007888.s001.tif]

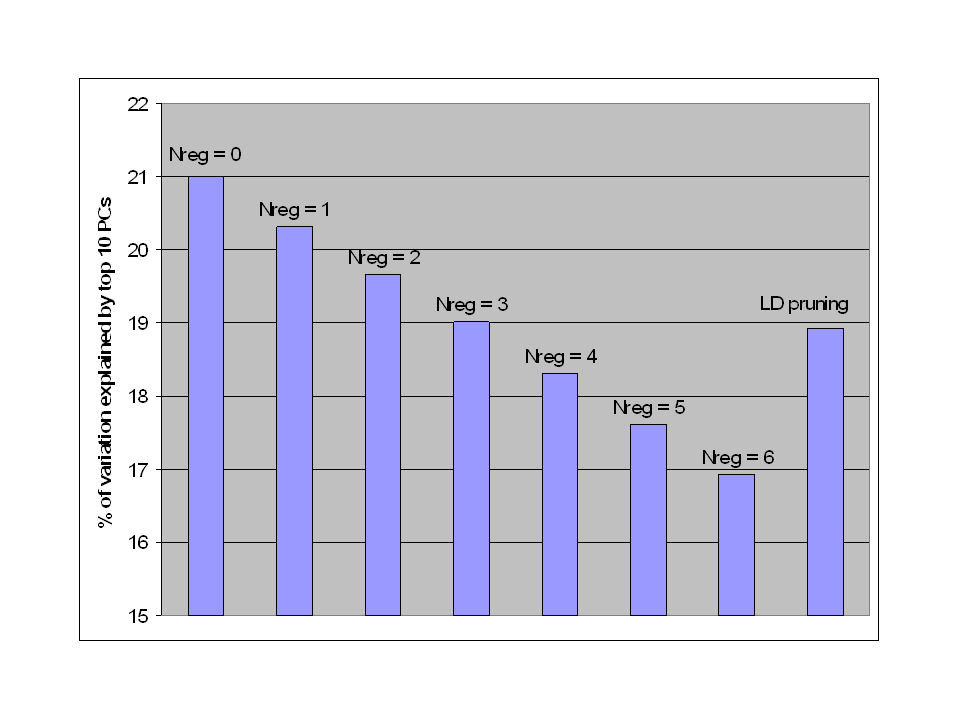

Supplement: Figure S2 — Effect of the number of preceding SNPs (Nreg) used to predict SNP genotypes in the regression analysis on the percent variation explained by the first 10 PCs. Also shown is the effect of pruning SNPs that are in high LD. (0.12 MB TIF) [file pone.0007888.s002.tif]

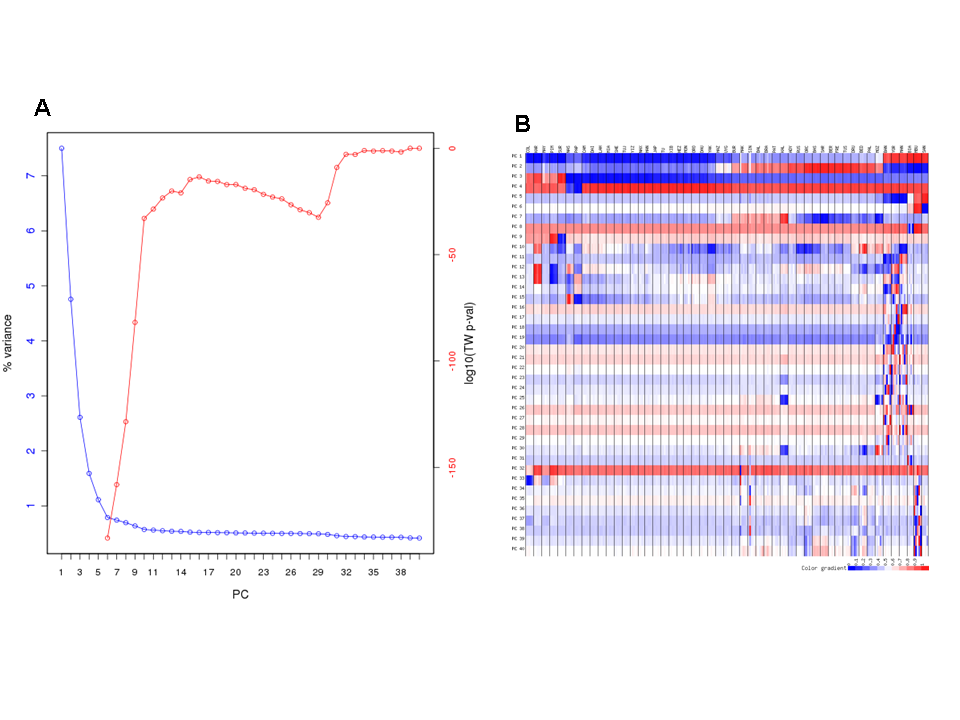

Supplement: Figure S3 — Analysis of additional PCs for the worldwide populations. (A) Amount of variation explained and associated statistical significance for the first 45 PCs. (B) Heat plot of the values of the first 45 PCs. The PC values have been normalized to range from 0 to 1. (0.44 MB TIF) [file pone.0007888.s003.tif]

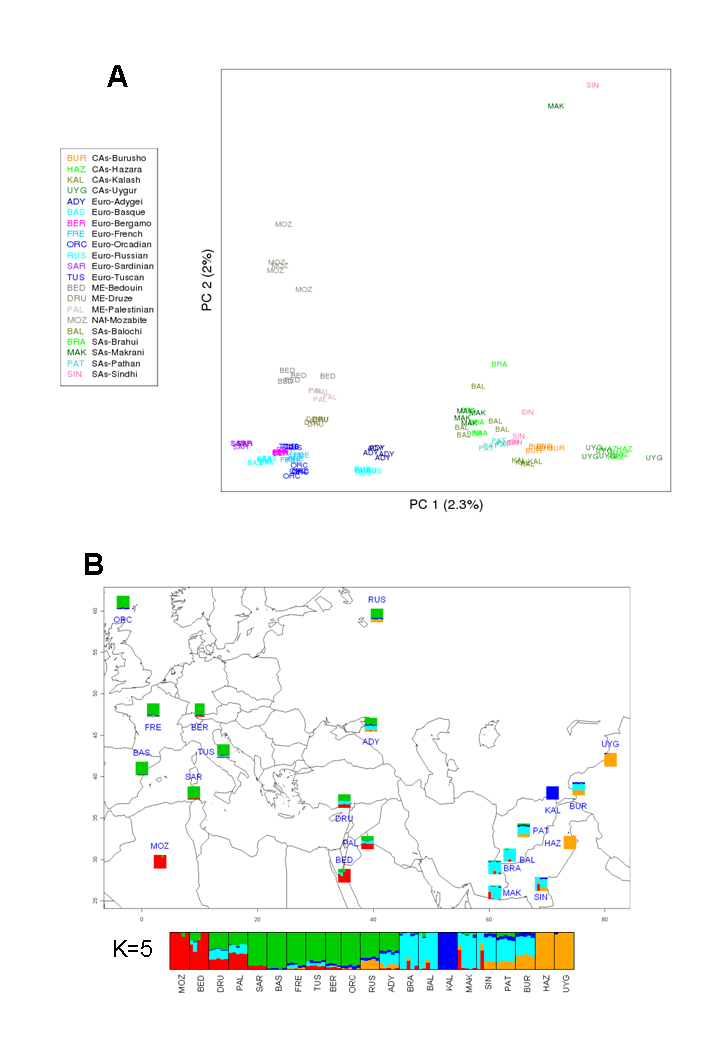

Supplement: Figure S4 — Regional analysis of the Europe, North Africa, Middle East, and Central/South Asia groups. (A) Plot of PC1 vs. PC2. (B) frappe results for K = 5. (0.19 MB TIF) [file pone.0007888.s004.tif]

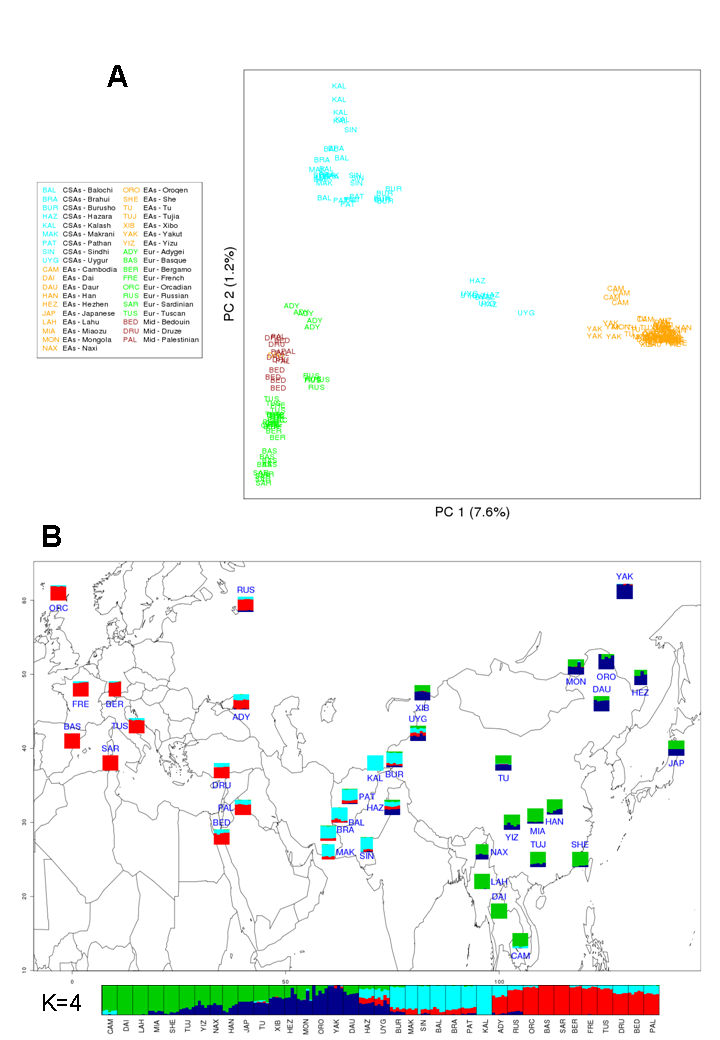

Supplement: Figure S5 — Regional analysis of the Europe, Middle East, Central/South Asia, and East Asia groups. (A) Plot of PC1 vs. PC2. (B) frappe results for K = 4. (0.24 MB TIF) [file pone.0007888.s005.tif]

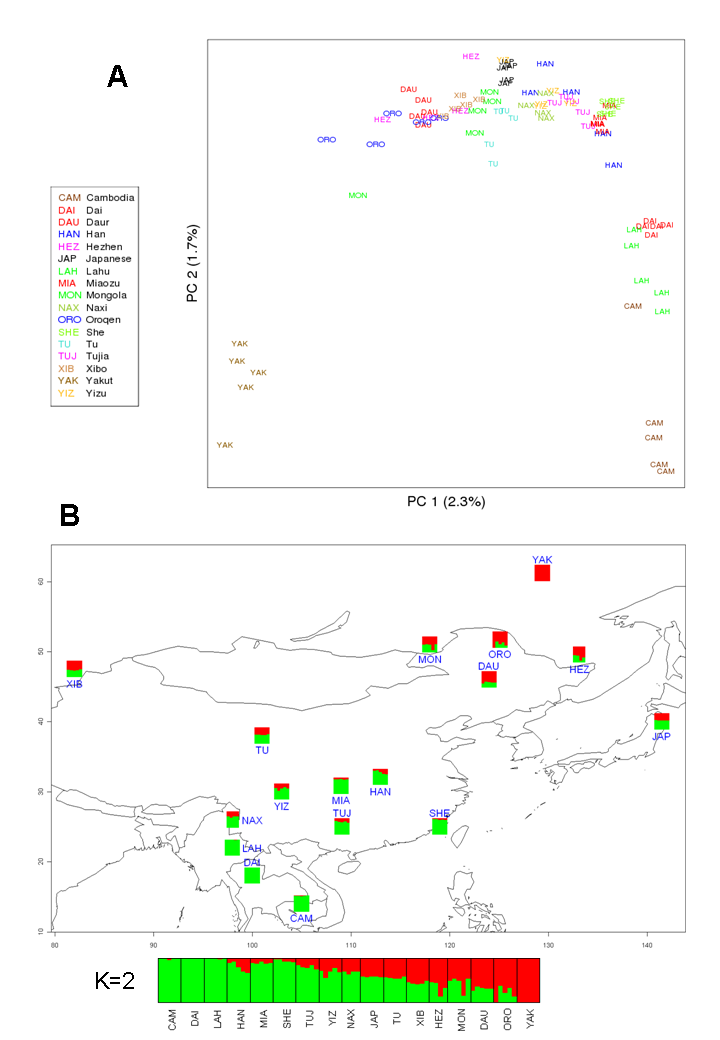

Supplement: Figure S6 — Regional analysis of the East Asian groups. (A) Plot of PC1 vs. PC2. (B) frappe results for K = 2. (0.17 MB TIF) [file pone.0007888.s006.tif]

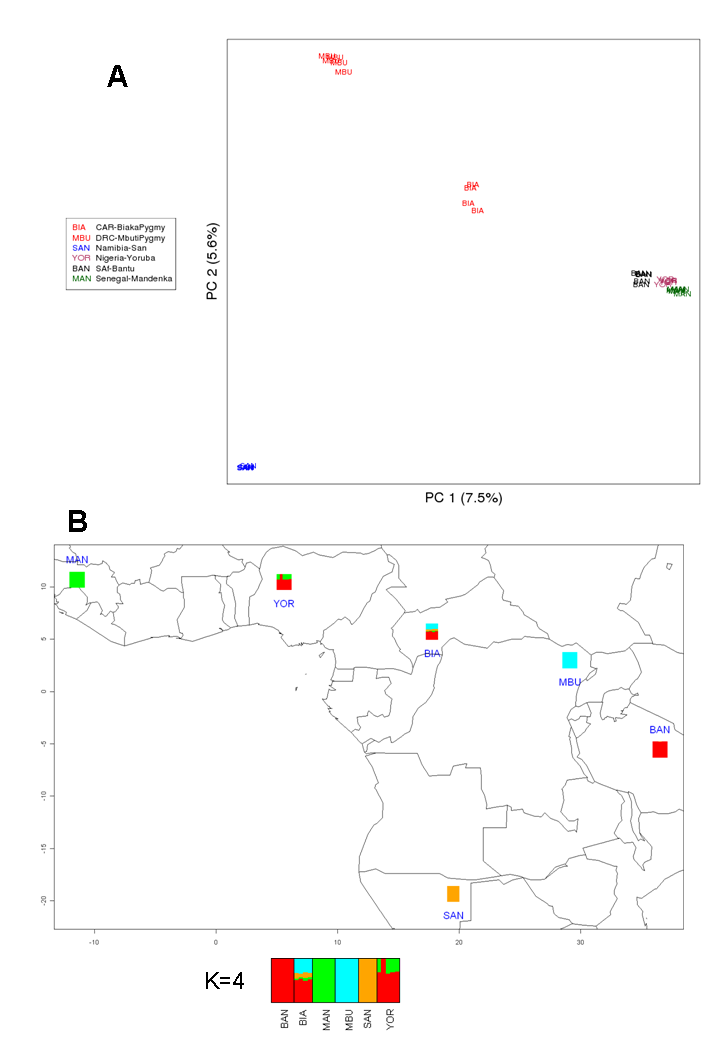

Supplement: Figure S7 — Regional analysis of the sub-Saharan African groups. (A) Plot of PC1 vs. PC2. (B) frappe results for K = 4. (0.12 MB TIF) [file pone.0007888.s007.tif]

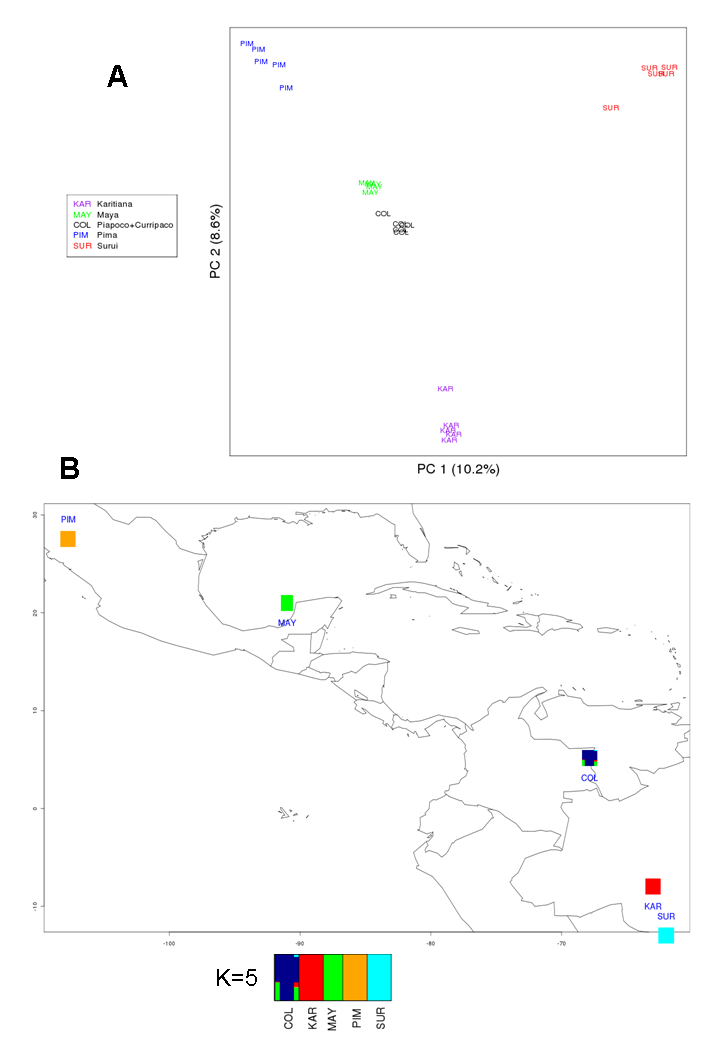

Supplement: Figure S8 — Regional analysis of the groups from the Americas. (A) Plot of PC1 vs. PC2. (B) frappe results for K = 5. (0.12 MB TIF) [file pone.0007888.s008.tif]

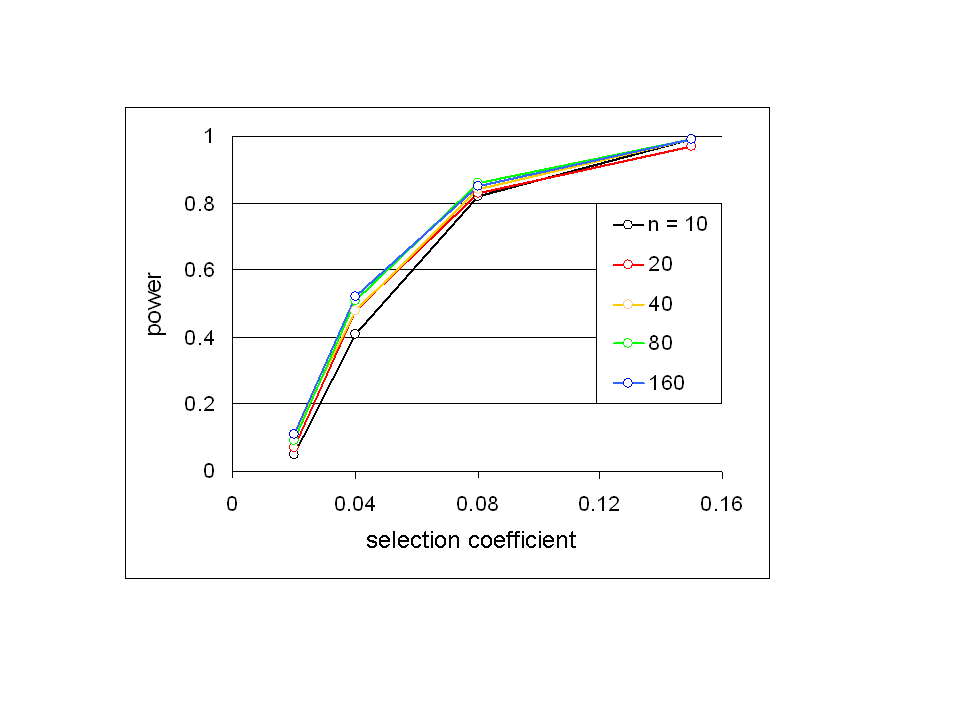

Supplement: Figure S9 — Effect of sample size on the power of the lnRsb analysis to detect a positively-selected allele. For each sample size (n = number of chromosomes), the fraction of simulations (y-axis) in which a selected allele of a given selection coefficient (x-axis) was detected as a candidate region of selection, according to the criteria described in the Methods section, is plotted. (0.06 MB TIF) [file pone.0007888.s009.tif]

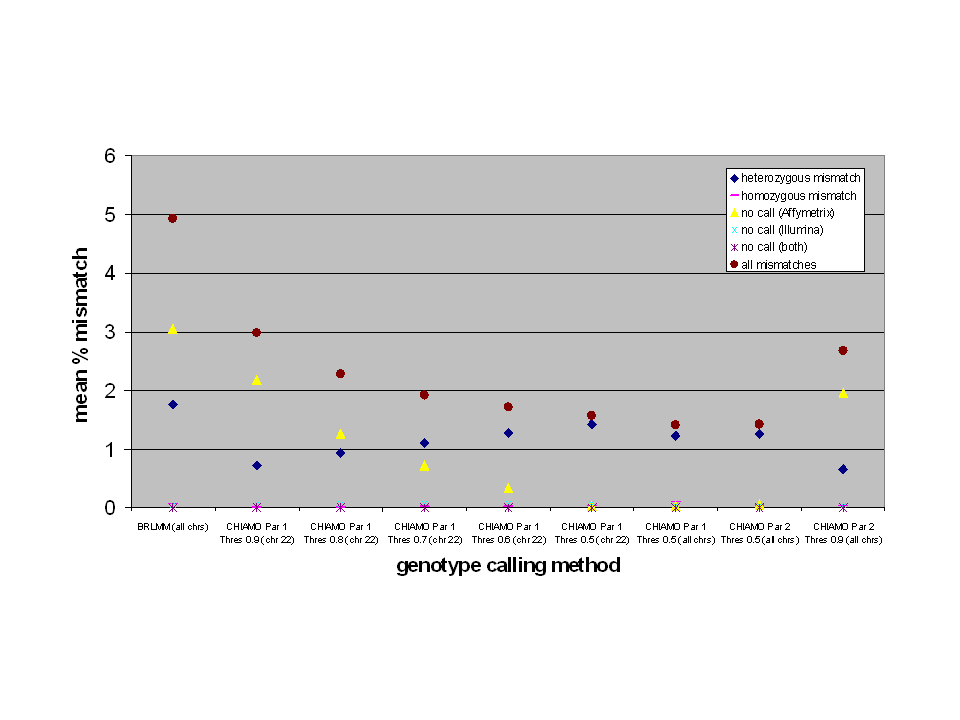

Supplement: Figure S10 — Effect of different parameter values on genotype calls obtained with the CHIAMO algorithm. Shown are the mean values (in percent) for heterozygous and homozygous mismatches between the Affymetrix and Illumina platforms, no calls for either or both platforms, and the sum of all mismatches and no calls. These values are given for the BRLMM algorithm for all chromosomes, and for two different parameter sets and various threshold values for the CHIAMO algorithm, for either just chromosome 22 or for all chromosomes. The threshold value is the maximum value for the total fraction of missing genotypes allowed; CHIAMO Par 1 is the WTCCC parameter set (-max1 -max2 -nmax 200 -n 0 -b 0 -f freqfile) and Par 2 is identical to Par 1 except without the -f option. (0.09 MB TIF) [file pone.0007888.s010.tif]
